# Supplementary material for: HIV treatment cascade among people who inject drugs in Ukraine
Source: PLoS One. 2020 Dec 31;15(12):e0244572. doi: 10.1371/journal.pone.0244572 (PMC7775055; doi:10.1371/journal.pone.0244572)
Supplement: S1 Table — (DOCX) [file pone.0244572.s001.docx]

*S1 Table. Characteristics of RDS recruitment in the study cities*

|  | **Sample size** | **Number of seeds** | **Number of waves** | **Recruitment homophily for HIV prevalence** | **Recruitment homophily for HIV status awareness** | **Recruitment homophily for self-reported ART uptake** | **Recruitment homophily for viral suppression** |
| --- | --- | --- | --- | --- | --- | --- | --- |
| Vinnitsya | 250 | 2 | 11 | 1.02 | 1.02 | 1.02 | 1.01 |
| Lutsk | 250 | 2 | 10 | 1.11 | 1.11 | 1.06 | 1.07 |
| Dnipro | 548 | 4 | 12 | 1.11 | 1.13 | 1.12 | 1.12 |
| Donetsk | 507 | 4 | 12 | 1.09 | 1.09 | 1.07 | 1.06 |
| Zhytomyr | 420 | 4 | 10 | 1.09 | 1.08 | 1.08 | 1.08 |
| Uzhgorod | 200 | 2 | 11 | 1.00 | 1.00 | 1.00 | 1.00 |
| Zaporizhzhya | 250 | 2 | 8 | 1.00 | 1.00 | 1.00 | 1.00 |
| Ivano-Frankivsk | 300 | 3 | 12 | 1.13 | 1.13 | 1.12 | 1.11 |
| Bila Tserkva | 350 | 3 | 12 | 1.11 | 1.13 | 1.13 | 1.10 |
| Kyiv | 350 | 3 | 11 | 1.05 | 1.05 | 1.07 | 1.05 |
| Kropyvnytskyi | 250 | 2 | 12 | 1.01 | 0.99 | 0.99 | 1.00 |
| Severodonetsk | 250 | 2 | 9 | 1.10 | 1.12 | 1.10 | 1.07 |
| Lviv | 403 | 3 | 14 | 1.10 | 1.08 | 1.08 | 1.07 |
| Mykolaiv | 500 | 4 | 13 | 1.08 | 1.08 | 1.07 | 1.08 |
| Odesa | 400 | 4 | 12 | 0.97 | 0.98 | 0.98 | 0.98 |
| Poltava | 401 | 3 | 12 | 1.07 | 1.09 | 1.08 | 1.06 |
| Rivne | 299 | 3 | 13 | 1.03 | 1.05 | 1.05 | 1.05 |
| Sumy | 200 | 2 | 8 | 1.02 | 1.01 | 1.01 | 1.01 |
| Ternopil | 248 | 2 | 10 | 0.99 | 1.00 | 0.99 | 0.99 |
| Kharkiv | 300 | 3 | 12 | 1.01 | 1.02 | 1.01 | 1.01 |
| Kherson | 350 | 3 | 10 | 1.05 | 1.04 | 1.04 | 1.07 |
| Khmelnytskyi | 250 | 3 | 9 | 1.04 | 1.04 | 1.04 | 1.04 |
| Cherkasy | 450 | 4 | 12 | 1.04 | 1.04 | 1.06 | 1.07 |
| Chernivtsi | 250 | 2 | 9 | 1.04 | 1.04 | 1.04 | 1.03 |
| Chernihiv | 400 | 4 | 12 | 1.31 | 1.32 | 1.29 | 1.29 |
| Sevastopol | 350 | 4 | 10 | 1.07 | 1.06 | 1.05 | 1.05 |
| Vasylkiv | 300 | 3 | 12 | 1.04 | 1.03 | 1.03 | 1.02 |
| Fastiv | 250 | 2 | 11 | 0.99 | 1.01 | 1.01 | 0.99 |
| Kryvyi Rih | 550 | 4 | 11 | 1.07 | 1.11 | 1.10 | 1.08 |
| Melitopol | 250 | 2 | 11 | 1.00 | 0.99 | 0.99 | 0.99 |
