# Supplementary figures and images for: HIV treatment cascade among people who inject drugs in Ukraine
Source: PLoS One. 2020 Dec 31;15(12):e0244572. doi: 10.1371/journal.pone.0244572 (PMC7775055; doi:10.1371/journal.pone.0244572)

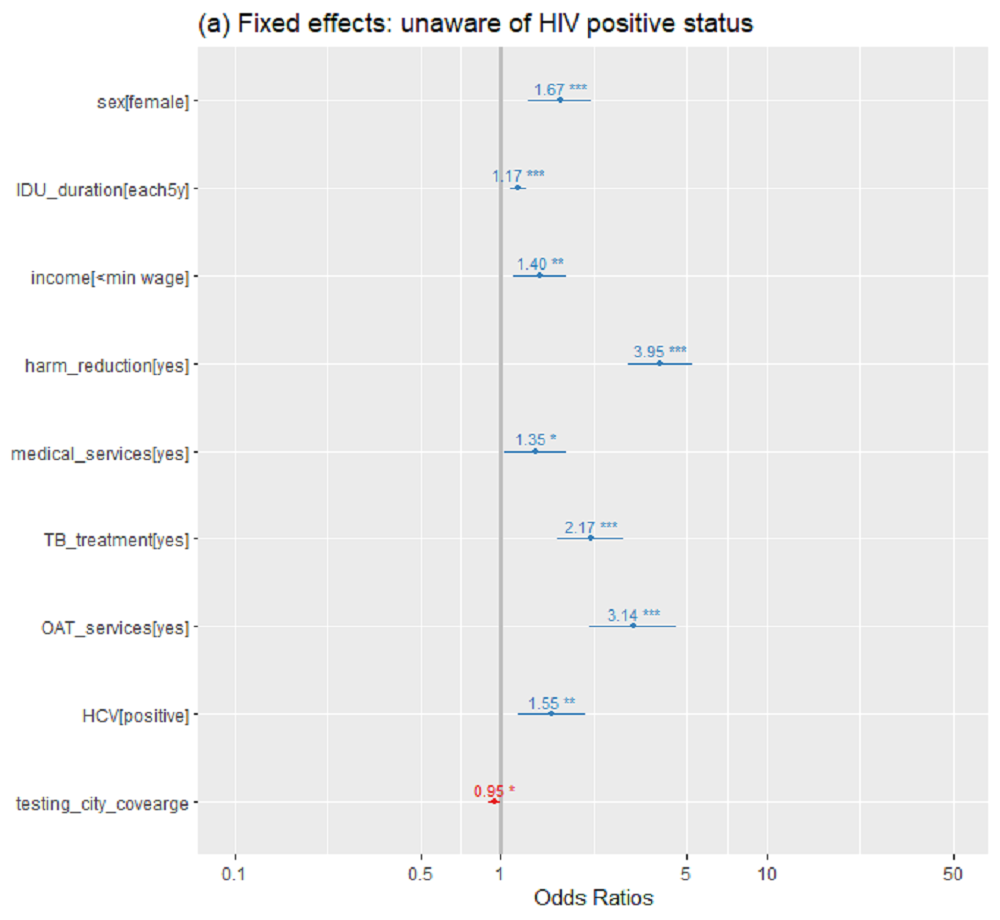

Supplement: S1 Fig — (TIF) [file pone.0244572.s002.tif]

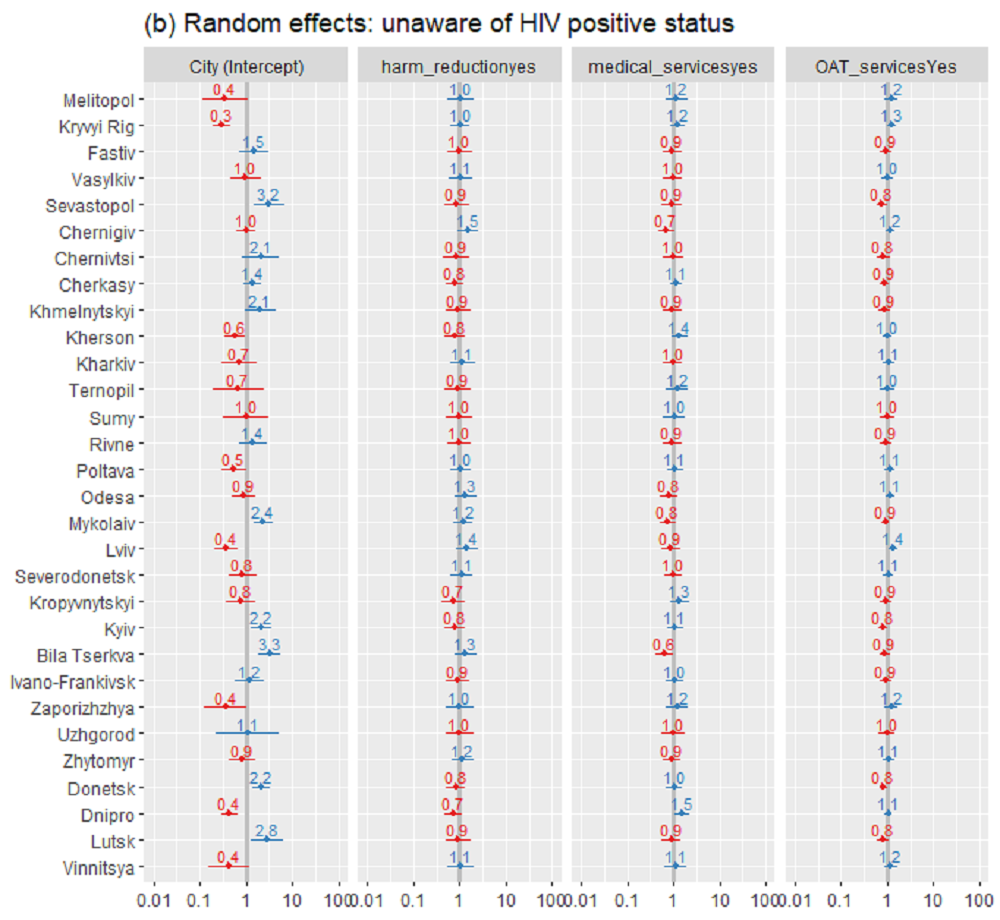

Supplement: S2 Fig — (TIF) [file pone.0244572.s003.tif]

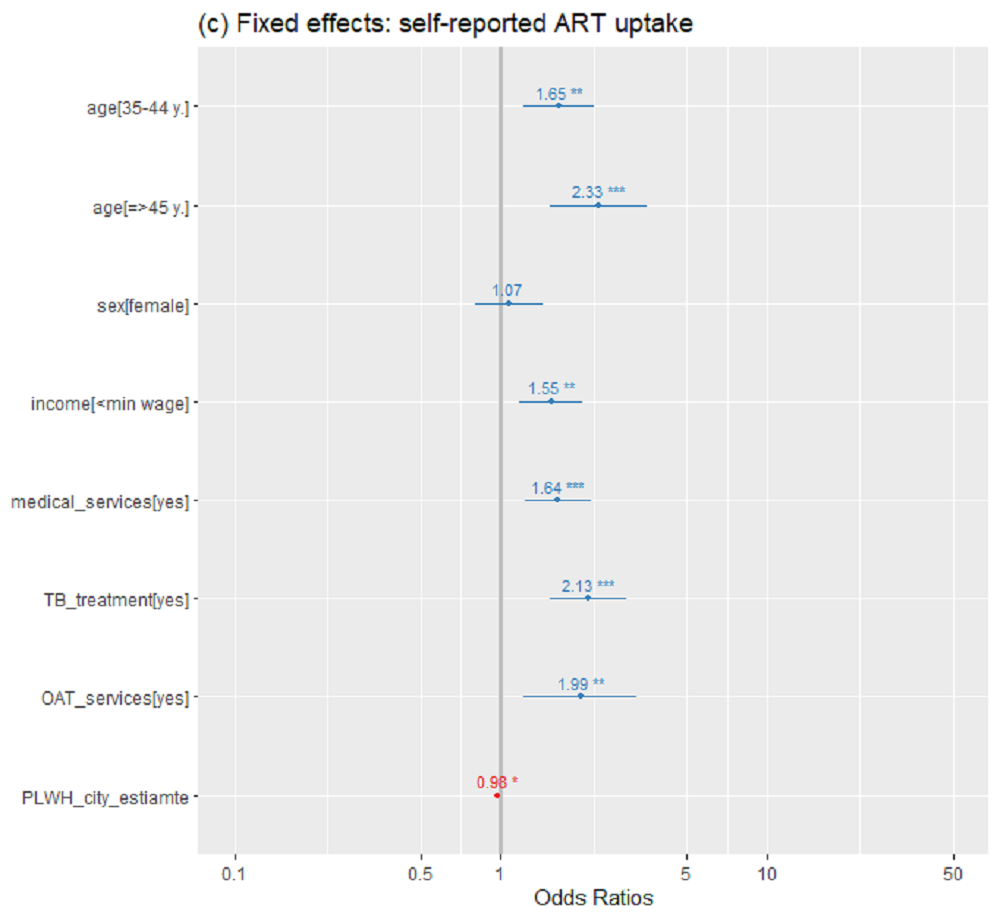

Supplement: S3 Fig — (TIF) [file pone.0244572.s004.tif]

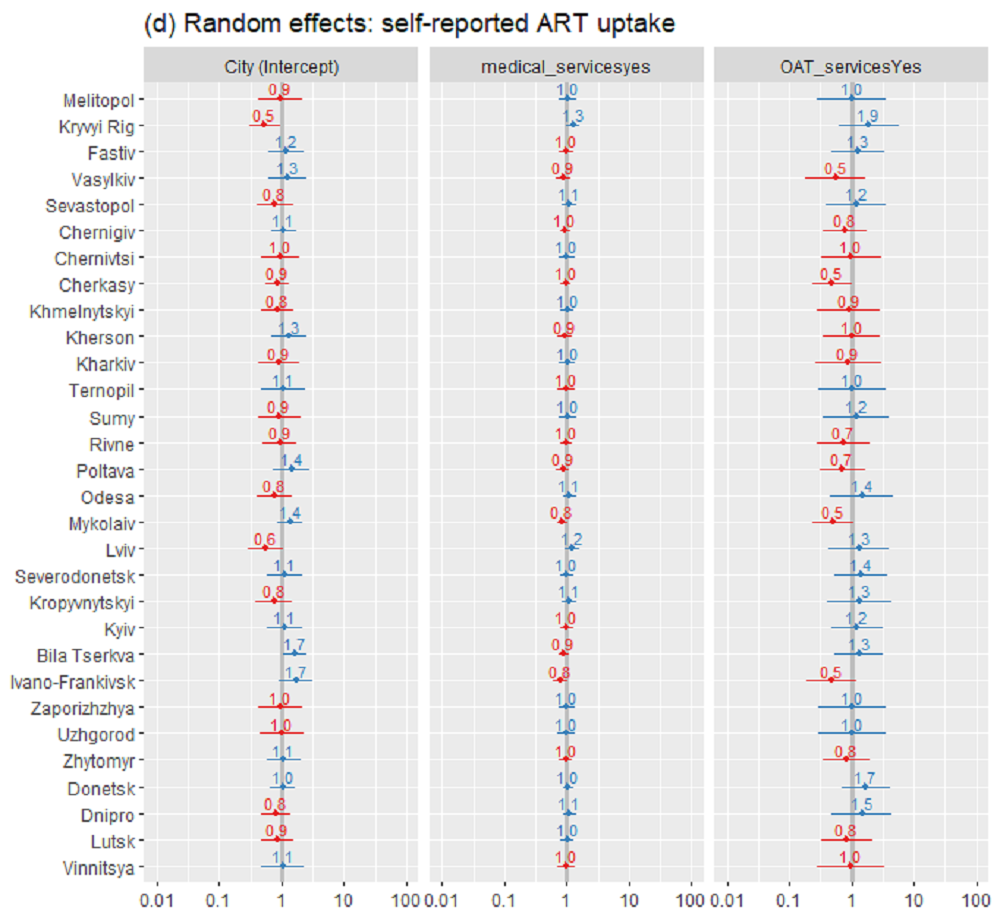

Supplement: S4 Fig — (TIF) [file pone.0244572.s005.tif]

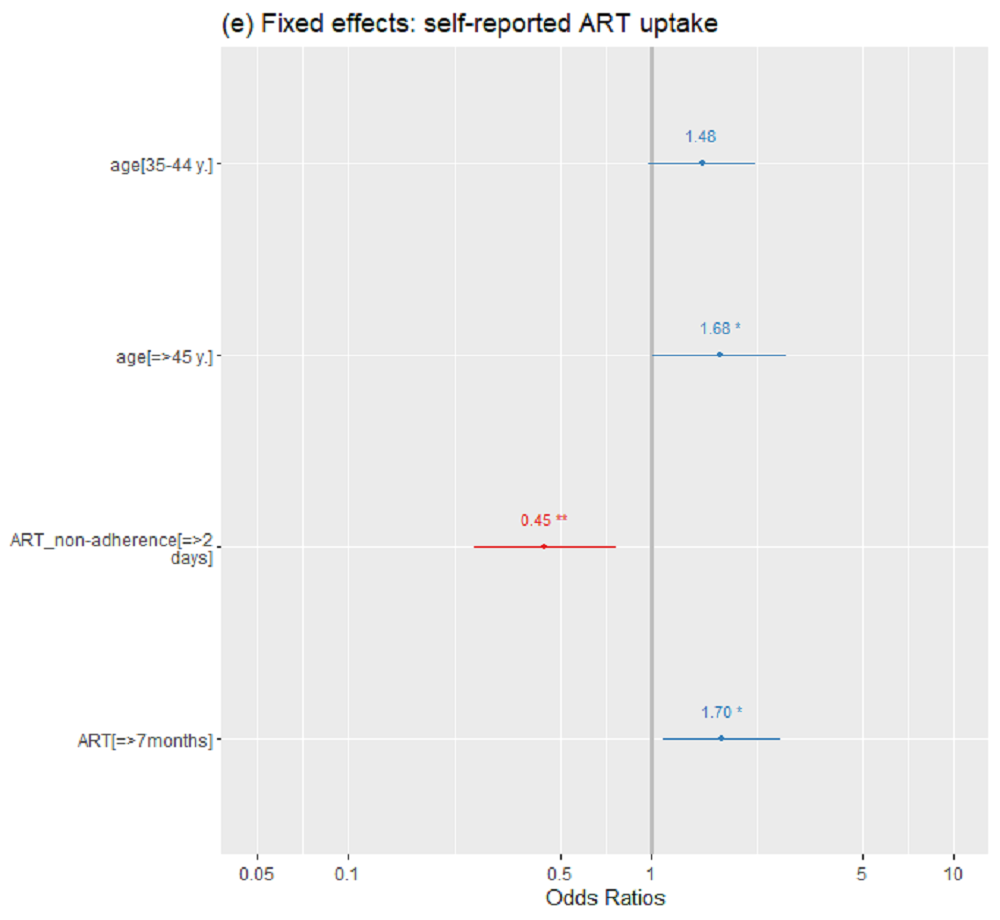

Supplement: S5 Fig — (TIF) [file pone.0244572.s006.tif]

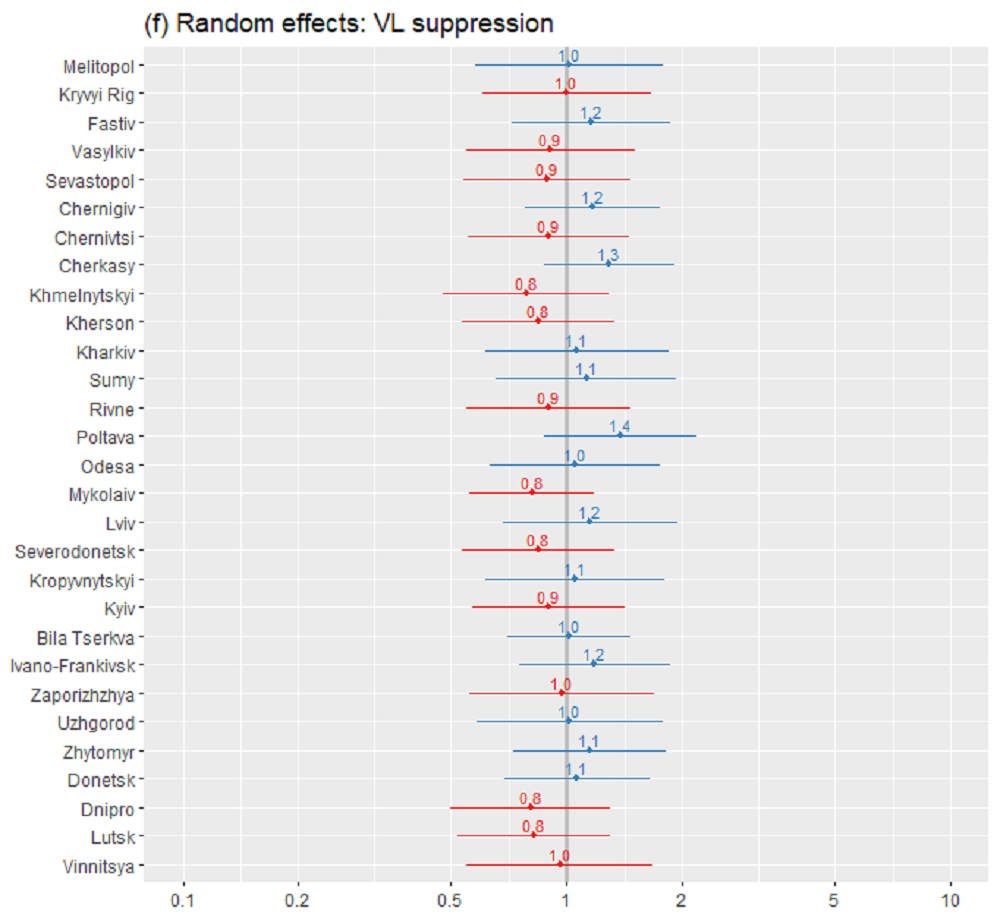

Supplement: S6 Fig — (TIF) [file pone.0244572.s007.tif]
